# Insights into Temperature and Hypoxia Tolerance in Cowpea Weevil via HIF-1

**DOI:** 10.3390/pathogens10060704

**Published:** 2021-06-05

**Authors:** Qin Liu, Zhichao Liu, Zhipeng Gao, Guanjun Chen, Changyan Liu, Zhenghuang Wan, Chanyou Chen, Chen Zeng, Yunjie Zhao, Lei Pan

**Affiliations:** 1 School of Life Sciences, Jianghan University, Wuhan 430056, China; lliuqin0316@jhun.edu.cn (Q.L.); gaozhipen@webmail.hzau.edu.cn (Z.G.); 201911110711121@stu.hubu.edu.cn (G.C.); ccy@jhun.edu.cn (C.C.); 2 School of Biological Information, Chongqing University of Posts and Telecommunications, Chongqing 400065, China; liuzc@cqupt.edu.cn; 3 Institute of Food Crop, Hubei Academy of Agricultural Sciences, Wuhan 430064, China; Liucy0602@163.com (C.L.); Zhwan168@163.com (Z.W.); 4 Department of Physics, The George Washington University, Washington, DC 20052, USA; chenz@gwu.edu; 5 Institute of Biophysics and Department of Physics, Central China Normal University, Wuhan 430079, China

**Keywords:** cowpea weevil, HIF-1α, mRNA transcript, inhibitor screening

## Abstract

Cowpea weevil (*Callosobruchus maculatus*) is a major pest that leads to severe damage of the stored leguminous grains. Several management approaches, including physical barriers, biological or chemical methods, are used for controlling bruchid in cowpea. These methods usually target the metabolically active state of weevil. However, it becomes less effective at early stages as egg, larva, or pupa under low temperature and oxygen conditions. Since hypoxia-inducible factor-1 (HIF-1) is known to coordinate multiple gene responses to low oxygen or low temperature signals, we examined the *HIF-1α* gene expression under low temperature and hypoxic treatments. At −20 °C, it took 4 h to reduce the survival rate for eggs, larvae, and pupae down to 10%, while at 4 °C and 15 °C, the survival rate remained higher than 50% even after 128 h as *HIF-1α* gene expression peaked at 15 °C. Moreover, HIF-1 protein offers a valuable target for early stage pest control complementary to traditional methods. In particular, HIF-1 inhibitor camptothecin (CPT), one of the five HIF-1 inhibitors examined, achieved a very significant reduction of 96.2% and 95.5% relative to the control in weevil survival rate into adult at 4 °C and 30 °C, respectively. Our study can be used as one model system for drug development in virus infections and human cancer.

## 1. Introduction

Cowpea weevil is a major pest of stored leguminous grains. The growth of cowpea weevil leads to severe damage to stored leguminous grains, such as cowpea, lentil, green gram, black gram, and other legumes [1,2,3]. Generally, storage losses due to cowpea weevil vary from 4% to 90% [4] and reduce the supply of major vegetable protein resources for humans in Asia and Africa [5]. Thus, it is essential to develop effective and safe methods to protect stored pulse grains against cowpea weevil.

The life cycle of cowpea weevil contains four stages: egg, four larval instars, pupa, and adult [6,7,8]. The adult lays eggs on the seed coat. Its larvae feed inside the grain seeds by eating the grain endosperm. These actions damage the seed viability and nutritional quality, making it unsuitable for replanting [1,9,10]. The physical barriers and chemical controls are widely used for controlling bruchid in cowpea (*Callosobruchus maculatus*) [3]. However, most current methods cannot kill the egg, four larval instars, and pupa [11].

Temperature management is one rational approach for pest control [12,13,14,15]. On the one hand, thermal stress has well-known detrimental effects on male fertility, affecting sperm competitiveness in cowpea weevil [16]. On the other hand, cowpea weevil has a different cold tolerance at different stages. The order of cold tolerance for cowpea weevil is eggs < adults < larvae < pupae at 0 °C. The developmental stages (eggs, larvae, pupae, and adults) of cowpea weevil could be controlled at −15 °C for 3 h [14].

Insects’ oxygen consumption offers another opportunity for pest control [17]. The low oxygen (hypoxia) manipulation has been applied in practice for the cowpea weevil control [18]. The average oxygen consumption is 8.3 mL per weevil from egg to adult in its life cycle [17]. Hypoxia stress can increase insect mortality, delay insect development, and disrupt metabolism [19].

HIF-1 protein can trigger and coordinate multiple gene regulation responses to low oxygen or low temperature signals [20,21,22]. HIF-1 protein is a heterodimeric DNA-binding complex, composed of two subunits: HIF-1α (an O_2_-labile α subunit, 120 kDa) and HIF-1β (a stable β subunit, 90 kDa) [21,23,24]. The mechanism underlying hypoxic response by HIF-1 protein has been widely investigated in mammals, birds, amphibians, fishes, and invertebrates [25,26,27,28,29,30]. A recent report on the hypoxic treatments of the cowpea weevil led to enhanced HIF-1 gene expressions, metabolic repression, and heat shock protein regulation [31]. To provide further evidence if HIF-1 protein can be targeted for pest control, we examined the HIF-1 gene expression under different temperature and hypoxia treatments. We analyzed the insecticidal activity of five HIF-1 inhibitors against cowpea weevil.

## 2. Results

### 2.1. Three Sub-Phases of Egg Development

We first examined the egg development of cowpea weevil under the stereomicroscope. The egg stage could be further divided into three distinctive sub-phases. In the early phase, the fertilized egg laid on a mung bean surface has an elliptical shape of size 0.6 × 0.4 mm^2^ with one end slightly squished and a shiny eggshell (Figure S1A). In the middle phase, the ovum begins to shrink from both sides to form a small vacuole. A black spot becomes visible as the contraction ended. The black area will develop into the larva head (Figure S1B). In the late phase, the blackhead of the larva is wholly formed (Figure S1C), and the eggshell finally becomes opaque with a cream white color from the frass deposits (Figure S1D), indicating the end of the late phase when the larva has burrowed into the bean.

### 2.2. The Survival Rate Effect upon Low-Temperature Treatment

**Figure 1:**
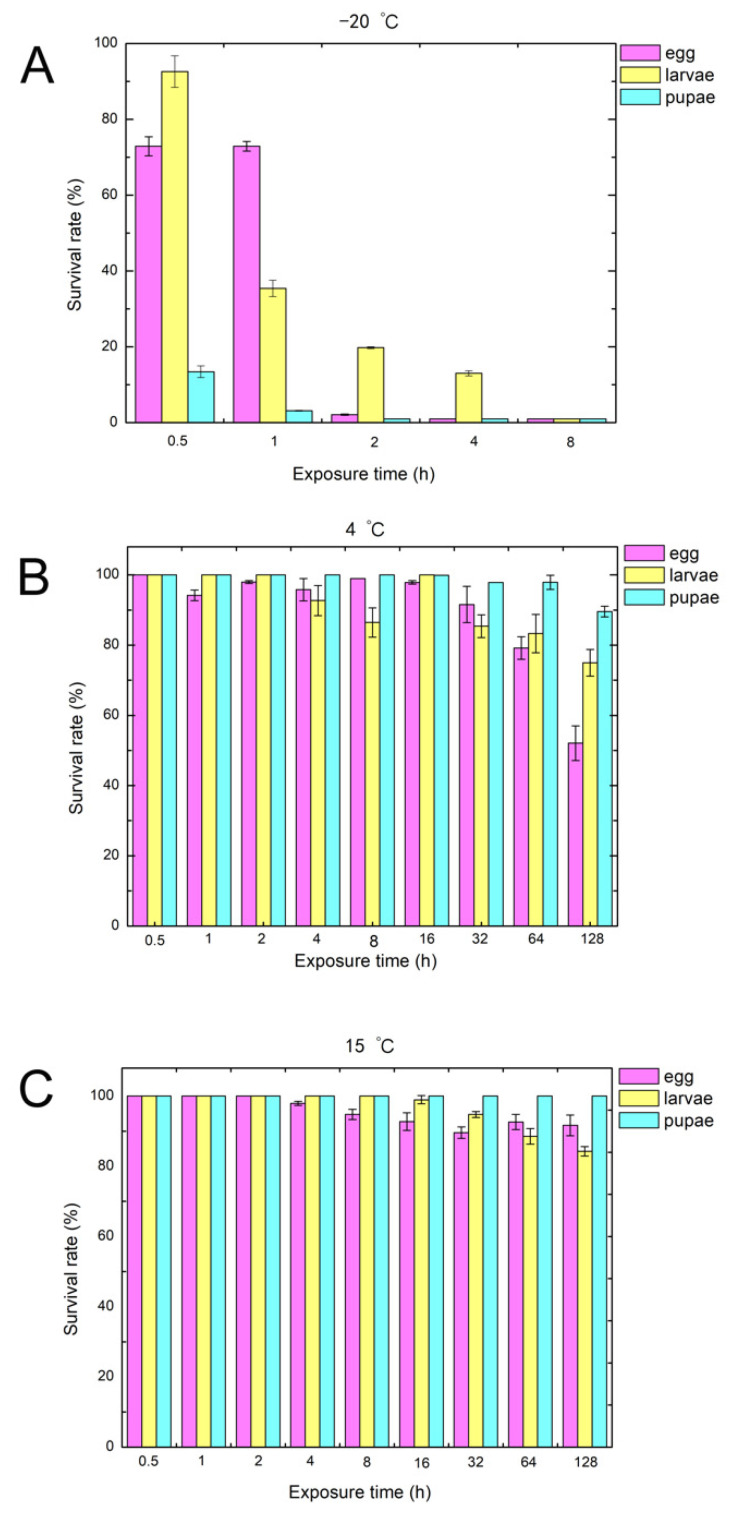
Effects of different low temperatures (from −20 °C to 15 °C) and different exposure times (from 0.5 h to 128 h) on the survival rates of eggs, larvae, and pupae. The subfigures (**A**), (**B**) and (**C**) represent the treatment at −20 °C, 4 °C, and 15 °C, respectively.

We examined the survival rate of weevils under different development stages upon exposure to three different temperatures of −20 °C, 4 °C, and 15 °C for different durations (Figure 1) [11]. The survival rate of the control group at 30 °C is 89%. More than 80% of pupae, 20% of eggs, and 7% of larvae were killed if exposed to −20 °C for 0.5 h (Figure 1A). It is noted that nearly 98% of eggs, larvae, and pupae were killed when the exposure time was extended to 8 h. The survival rates of eggs, larvae, and pupae stayed at a relatively high level at 4 °C (Figure 1B). About 50% of eggs, 25% of larvae, and 10% of pupae were killed when the exposure time was extended to 128 h. In contrast, the higher temperature of 15 °C had little effect on the cowpea weevils at any stage of development. Less than 10% of eggs, 15% of larvae, and none of the pupae were killed even when the exposure time was extended to 128 h (Figure 1C). These results showed that exposure to the temperature of −20 °C significantly diminished the survival rate of cowpea weevils, while exposure to low temperatures of 4 °C and 15 °C had little impact. To gain insights into these observations, we measured the expression level of hypoxia-inducible factor-1α (HIF-1α) since it is expected that HIF-1 protein can coordinate a multiple-gene response to counter exposures to mild low temperature or low oxygen.

### 2.3. Sequencing of Cowpea Weevil HIF-1α cDNA

**Figure 2:**
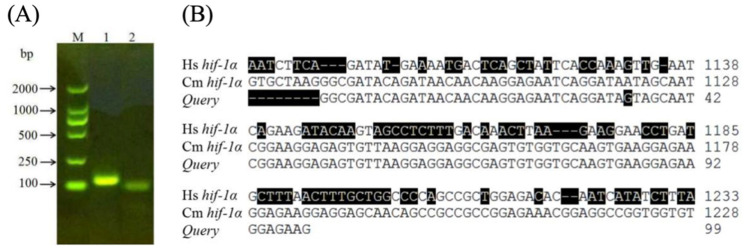
(**A**) RT-PCR products amplified by different primers. Lane M: DL 2000 DNA marker; Lane 1: the fragment product with primers 18S-F and 18S-R for 18S rRNA as control; Lane 2: the fragment product with primers *1α-F* and *1α-R* designed to amplify a 96-bp fragment of *Cm HIF-1α* gene; (**B**) The actual query nucleotide produced in Lane 2 of (**A**) above was sequenced. It showed a very high sequence similarity to *Cm HIF-1α* gene as expected (GenBank accession number JN228344) and a low sequence similarity to *Hs HIF-1α* in *Homo sapiens* (GeneID:3091). Nucleotides different from the reference *Cm HIF-1α* DNA sequence are shaded black.

It is known that many hypoxia-sensitive mammalian organs activate multiple genes to restore energy and oxygen homeostasis at low temperature or low oxygen levels [32]. Such an adaptation utilizes the HIF-1 protein in the glycolytic pathway for energy production and stimulates angiogenesis and erythropoiesis to increase tissue oxygenation. To probe the changes in *HIF-1α* gene expression under different temperature conditions, the insects were exposed to various temperatures (4 °C, 15 °C, 30 °C, and 37 °C) in our study. One specific amplification band (~100 bps) was obtained in the RT-PCR experiment (Figure 2A, Lane 2) using primers 1α-R and 1α-F designed to amplify a 96-bp fragment of *Cm HIF-1α* cDNA. Indeed, the sequence measured for this product shared a very high sequence similarity of 99% to the *Cm HIF-1α* cDNA sequence (GenBank accession number JN228344) and a lower sequence similarity of 60% to *Hs HIF-1α* gene in *Homo sapiens* (GeneID: 3091).

### 2.4. Relative Expression of HIF-1 Gene under Temperature and Oxygen Treatment

**Figure 3:**
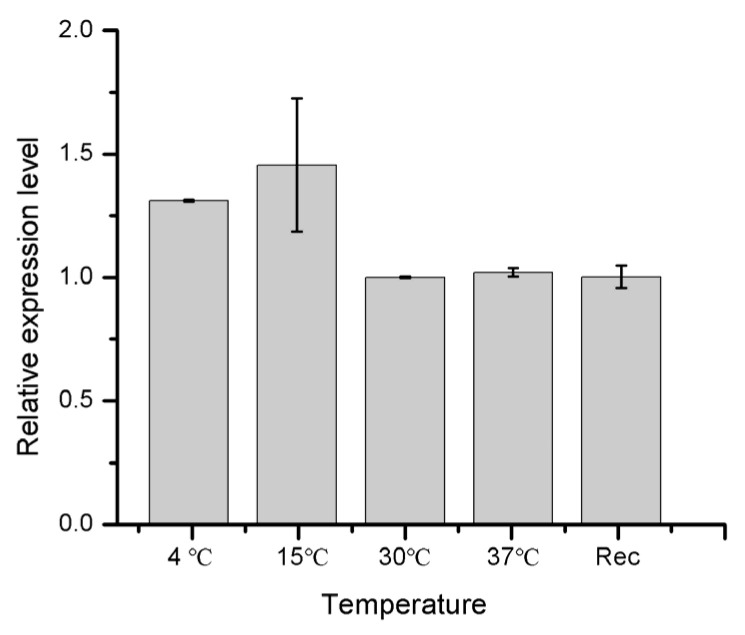
The relative expression level of *HIF-1*α gene in 4th instar larvae of cowpea weevils under different temperature treatments. The larvae incubated at the 30 °C were subjected to different temperatures indicated above for 24 h before RNA extraction. Rec here denotes a procedure where the 24 h exposure to 4 °C was followed by another 24 h recovery under 30 °C before RNA extraction.

Using the RT-PCR technique, we further measured the differential expression of *HIF-1*α mRNA transcript in the 4th instar larval stage of cowpea weevils under different temperature and oxygen treatments. Figure 3 shows the relative expression level of the *HIF-1*α gene when the larvae were exposed to low temperatures of 4 °C and 15 °C, and room temperatures of 30 °C and 37 °C for 24 h. The Rec stands for the treatment of 4 °C for 24 h followed by a recovery of 24 h at 30 °C. The *HIF-1*α gene shows an elevated expression of about 1.5 folds at low temperatures compared to room temperatures. The considerably large fluctuation in expression for biological replicates observed at 15 °C is suggestive that 15 °C may be a transition point for HIF-1 protein adaptation where HIF-1 protein oscillates between on and off.

**Figure 4:**
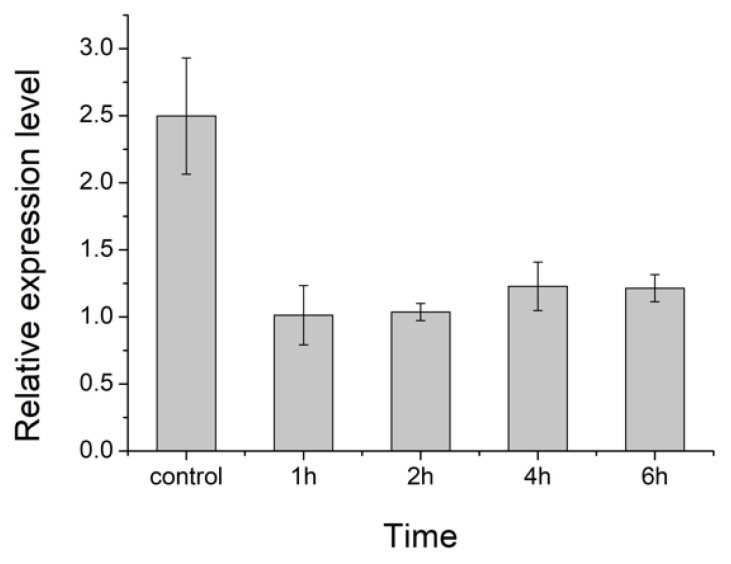
The relative expression level of *HIF-1*α gene in 4th instar larvae of cowpea weevils under presumably different oxygen levels. Control here denotes the procedure where RNA extraction was immediately performed when live larvae were taken out from the beans. Other treatments are marked by the duration of hours that the live larvae taken out from the beans were further exposed to the ambient air before RNA extraction.

We also measured the relative expression level of the *HIF-1*α gene in the 4th instar larvae of cowpea weevils under 30 °C when they were taken out of the beans to be exposed to ambient air mimicking presumed reoxygenation for different durations (Figure 4). The significant decreases in *HIF-1*α expression once exposed to oxygen-rich air strongly suggest a hypoxia condition typically encountered by larvae inside the bean where *HIF-1* gene regulation is required and activated even at these early stages. Together, these results offer an opportunity to use HIF-1 inhibitors to shut down the early life cycle of cowpea weevils.

### 2.5. Reduction in Survival Rate of Cowpea Weevils upon HIF-1 Inhibitor Treatments

To examine whether HIF-1 protein can be a viable target for pest control, in particular, at the early stages of weevil’s life cycle where the development takes place without exposure to the ambient air and presumably in a hypoxia environment, we screened five HIF-1 inhibitors (2ME2, CPT, TPT, VCR, and PTX) for their effects on cowpea weevil’s life cycle under two different temperature settings (see Section 4.7 for experiment procedures). The experimental results on the survival rate of cowpea weevils, which is defined as the fraction of emerging adults, are summarized in Table S1.

**Figure 5:**
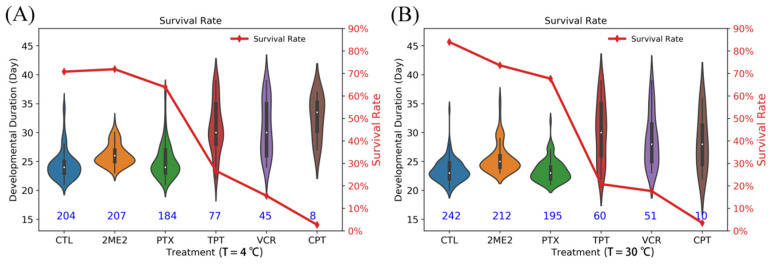
The survival rate and developmental duration of cowpea weevils from the initial egg stage to the final adult stage under two different incubation temperature settings of 4 °C (**A**) and 30 °C (**B**). Treatments under five different HIF-1 inhibitors are marked as 2ME2, PTX, TPT, VCR, and CPT. CTL represents the control sample. The five inhibitors are: 2-methoxyoestradiol (2ME2), vincristine (VCR), camptothecin (CPT), topotecan (TPT), and paclitaxel (PTX).

Compared to the survival rate of 84.0% for the control (242/288), 2ME2 and TPT led to comparable survival rates of 73.3% and 67.6%, respectively, representing only 12.0% and 19.5% reductions, on the other hand, PTX, VCR, and CPT led to much lower survival rates of 20.8%, 17.7%, and 3.5%, respectively. In particular, the lowest survival rate of 3.5%, i.e., a 95.5% reduction relative to the control (1–3.5%/84.0% = 95.5%), demonstrates the remarkable efficacy of CPT to stop weevils from completing the life cycle into the final adult stage. Interestingly, as shown in Figure 5, the two less effective inhibitors (2ME2 and TPT) as well as the control showed a clearly peaked distribution with a typical hatching time centered at 22–24 d. In contrast, the more effective inhibitors (PTX, VCR, and CPT) all led to a conspicuously broad and flat distribution without a clearly defined peak on hatching time, suggesting that these inhibitors completely alter the normal development process with a chaotic biological clock instead of a delayed clock with a shifted peak. While the detailed mechanism remains to be elucidated, it is suggestive that such a disorderly clock of development could have a more detrimental effect on the adult weevils than the low survival rate of 3.5% would already suggest.

Since HIF-1 protein coordinates the response to both low oxygen and low temperature signals, we also examined the potential benefits of HIF-1 inhibition in further reducing the survival rate of weevils at a lower temperature. To do so, we modified the above experiment by lowering the incubation temperature of 30 °C to 4 °C for only 24 h between the 15th and 16th day. The results are shown in Table S1 and Figure 5. Indeed, the survival rate for the two most effective inhibitors of VCR and CPT was slightly further reduced to 15.6% and 2.7%, respectively. Here, 2.7% represents a 96.2% reduction from that of the control, which had a survival rate of 70.8% (1–2.7%/70.8% = 96.2%).

Taken together, our results demonstrated that HIF-1 inhibitor CPT could achieve a significant reduction of about 95% in weevil’s survival rate of the controls at either room temperature or storage room temperature.

## 3. Discussion and Conclusions

Insects have a certain degree of adaptability to the low temperature stress. The insects can synthesis the antifreeze protein to prevent the growth of ice crystals and avoid permanent damage to cells and organs [33]. The previous investigation indicated that the C. maculatus eggs showed the cold-tolerant among five store-product insects. The LD50 values of the C. maculatus eggs were 2.7, 1.3, and 0.3 h at −10 °C, −15 °C, and −20 °C, respectively [34]. Among the different developmental stages of C. maculatus, pupae showed the best tolerance under and cold treatment of 0 °C [35].

The standard chemical methods of controlling storage pests concentrate on developing effective fumigation and aeration protocols [32]. However, it is difficult for these methods to stop the larvae of cowpea weevils residing inside the beans [14]. The chemical residues retained on the fumigated beans could also promote chemical resistance [36,37]. Other physical methods utilizing low temperature and/or low oxygen conditions have also been explored to disrupt the life cycle of cowpea weevils [7,14]. For example, cold air can be blown into the warehouse to slow the growth or kill the insects [14]. However, the cowpea weevils can adapt to environmental stress, for example, by activating such a master regulator as HIF-1 protein and recover back at normal conditions [31,38].

ATP is the primary source of metabolic energy for insects. The cold temperature or hypoxia conditions affect the insect’s metabolic energy. Thus, the insects have developed the HIF-1 to respond to the temperature and hypoxic [39,40]. Interestingly, for the cowpea weevils we studied, the HIF-1 response was already activated even with the temperature of 15 °C or presumably mild hypoxia interior of the mung bean [32,41,42]. This makes HIF-1 an attractive target without maintaining a cold storage room and the associated energy consumption. Here, we investigated the survival rate and developmental duration of cowpea weevils upon HIF-1 inhibitors [43,44,45,46,47]. One specific HIF-1 inhibitor CPT was found to reduce the survival rate of cowpea weevils by a remarkable 95.5% even at room temperature (30 °C). This HIF-1 inhibitor approach will be particularly useful in the industry where fumigation with methyl bromide has become almost one standard tool for the dried beans protection [41].

There is a noticeable positive correlation between the low survival rate and the broad and uniform distribution of hatching time. This is strongly suggestive that weevil’s biological clock of its development cycle was not merely delayed but disrupted. It remains to be investigated if such a disruption becomes detrimental to the survived adults or their offspring, making the inhibitor more effective than it already is. The diverse effects of these putative HIF-1 inhibitors targeting different stages of HIF-1 activation remain elusive. Further studies are needed to elucidate the molecular mechanism of inhibition on weevil’s life cycle and search for more potent compounds.

Regardless of the detailed mechanism, the high efficacy of CPT in reducing survival rate by 95.5% and 96.2% at 30 °C and 4 °C, respectively, with a simple application scheme of mixing the compound powder with mung beans serves as a proof-of-concept in targeting HIF-1 for weevil control. Quantitative and systematic examinations on the scheme and/or schedule of applying these inhibitor compounds to the beans will further optimize their efficacy. The HIF-1 can also trigger and coordinate multiple genes’ up-regulation in virus infections and human cancer. Our study can be used as one model system for drug development in HIF-1 related human diseases.

## 4. Materials and Methods

### 4.1. Insect Source and Feeding Method

Samples of *C. maculatus* were provided by the Institute of Food Crops, Hubei Academy of Agricultural Sciences, China. All cowpea weevils were reared in a smart chamber (HP1000GS-B, Shanghai Jingsheng Scientific Instruments Co., Ltd., Shanghai city, China). Adult weevils were released into a weevil-free chamber containing sterilized and dried mung beans to lay eggs on the beans. These inoculated beans were then placed in a glass dish covered with a perforated plastic wrap at an incubation temperature of 30 ± 0.5 °C and relative humidity of 70 ± 5% with photoperiod 12L:12D [38]. Weevils at different stages of development were harvested from these beans for various tests as described below. Active adults were selected to repeat the procedure to maintain a continuous colony.

According to the previous reports [38] and our trial runs, the three main stages in the life cycle of a cowpea weevil were observed as the following: the egg stage from first day (d) to 5th d, the larval stage from 6th d to 15th d, and the pupal stage from 16th d to 21th d. Adults within 24 h of emergence were used to inoculate beans for 24 h for well-formed eggs.

To obtain full eggs of cowpea weevil consistently, sub-phases of the egg stage were carefully inspected under microscope. For this study, the weevils between first d and second d, 10th d and 11th d, and 17th d and 18th d, were selected as the experimental materials to represent egg, larval, and pupal stage, respectively, for subsequent tests and analyses.

As a control to study the effect of low temperature on cowpea weevil’s development, the entire developmental cycle of cowpea weevil was first investigated at 30 °C and relative humidity of 70 ± 5%.

### 4.2. Observation of Egg Morphology

Since the whole larval stage of the cowpea weevil happens inside the bean seed, the transition from the egg stage to the larval stage was studied. The single-grain eggs (egged mung beans) produced in the first 24 h by emerging adult female weevils were incubated in a 30 °C climate chamber, and the morphology of the eggs was observed and recorded by a stereomicroscope every 12 h. When a hatched larva completely entered the bean, it was considered that the larva successfully completed the egg stage.

### 4.3. The Effect of Low Temperature on the Survival of Cowpea Weevils at Different Stages of Development

96 egg-attached mung beans of the first day were treated in a 96-well plate at three different temperatures (−20 °C, 4 °C, and 15 °C) for 9 different durations (0.5, 1, 2, 4, 8, 16, 32, 64, and 128 h). The group maintained at temperature 30 °C was used as the control. Three biological replicates were performed for both the control and low temperature treated groups. To define the end of the larval stage, a clear circular spot on the husk is visible under the stereomicroscope [32]. The biting of a mature larva causes this thin circular hole cover of about 2 mm in diameter before the onset of its pupation.

At the larval stage, 50 egg-attached mung beans were chosen that were between 10 d to 11 d after the eggs were laid. The color of eggshells at this stage became cream white under microscope indicating normal development of larvae inside the bean. These larvae were similarly treated in a 96-well plate at three different temperatures for eight different durations as in 4.3.1 followed by incubation afterwards at 30 °C until pupation. The percentage of completed pupations under different treatments were recorded and compared with that of the control group maintained constantly at 30 °C. Three biological replicates were performed.

At the pupal stage, 50 egg-attached mung beans between the 17th d to 18th d were selected for the low-temperature treatment. They were treated in a 96-well plate at three different temperatures for eight different durations followed by incubation at 30 °C to record the successful rate of adult weevils emerged from the beans and compare the rate with that of control group at constant 30 °C. Three biological replicates were used.

### 4.4. Extraction of Total RNA under Different Temperature and Oxygen Treatments

First, the mung beans with eggs were incubated at 30 °C for 14 days. The 14th day eggs correspond to the 4th period of larval stage [41]. We divided mung beans with eggs into two parts. Then, the mung beans with larvae were subjected to different temperature and oxygen treatments.

For the temperature treatments, the mung beans with larvae were further exposed to 4 °C, 15 °C, 30 °C (as control since no temperature change), and 37 °C for 24 h before RNA extraction. Rec here denotes a procedure where the 24 h exposure to 4 °C was followed by another 24 h recovery under 30 °C before RNA extraction. For different reoxygenation treatments, live larvae in the beans were carefully taken out and exposed to normal oxygen condition for 1 h, 2 h, 4 h, and 8 h before RNA extraction. Control here denotes the procedure where RNA extraction was immediately performed when live larvae were taken out from the beans.

As for total RNA extraction, the larvae in the beans were carefully removed with tweezers and frozen with liquid nitrogen either immediately or after a specified duration of exposure to the ambient air. The total RNA was extracted following the instructions of the Trizol-based method (Invitrogen, USA). The purity and concentration of the total RNA were estimated with Qubit 2.0 Fluorometer (Life Technologies).

### 4.5. Design and Synthesis of Primers and Gene Sequencing

HIF-1α is respond to trigger and coordinate multiple gene regulation under low oxygen or low temperature signals. To identify the target gene *HIF-1α,* the PCR amplification and sequencing were carried out as the following steps. After total RNA was treated with DNase I (Promega, Madison, USA), cDNA was synthesized by reverse transcription according to the instructions of PrimeScript^TM^ 1st Strand cDNA Synthesis Kit (Takara, Takara Biomedical Technology (Dalian) Co., Ltd., Dalian city, China) and stored at −20 °C. Primers for the target gene *HIF-1α* were designed using the online software OligoArchitectTM Online (http://www.oligoarchitect.com/, accessed on April 2020) that resulted in forward primer 5′-GGCGATACAGATAACAACAA-3’(1α-F) and reverse primer 5′-TCTCCTTCTCCTTCACTTG-3′ (1α-R). The amplified fragment size was of 96 bps for the target gene.

18S rRNA was used as an internal reference gene in RT-PCR [31]. The sequences of the forward and reverse primers were 5′-ATCACGGTGCTCTTTACT-3′ (18S-F) and 5′-CGAGATCCTATTCCATTATTCC-3′ (18S-R), respectively. The amplified product fragment was of 124 bps by the two 18S rRNA primers. Both primer synthesis and gene sequencing measurement were performed by Wuhan Qingke Innovation Biotechnology Co., Ltd.

### 4.6. Standard Curve, RT-qPCR, and Data Analysis

Expression analysis of the target gene *HIF-1α* in cowpea weevil was carried out by semi-quantitative RT-PCR, using 18s rRNA as an internal control. *HIF-1α* gene was selected to evaluate the transcript response to different temperatures and oxygen treatments.

Five concentration gradients of cDNA templates were prepared by diluting with bi-distilling water at 1:2 (*v*/*v*). A standard curve was obtained using each concentration gradient of cDNA as template. Three biological repeats were applied for each concentration gradient of cDNA.

The RT-PCR reaction was performed using the ABI 7900 HT system (Applied Biosystems). A total of 20 μL-reaction volume included 1 μL cDNA template, 1 μL forward and reverse primers (2 μM), 10 μL 2 × SYBR Green Master Mix (Bio-Rad), and 8 μL sterilized water. The amplification reaction conditions were as follows: pre-denaturation at 95 °C for 5 min, denaturation at 95 °C for 10 s, annealing at 59 °C for 30 s, extension at 72 °C for 20 s, 40 cycles in total, and dissolution curve at 54–95 °C for amplification products. The amplified fragments were separated in 2% agarose by gel electrophoresis. The relative expression of the target gene *HIF-1α* was calculated using the 2^-ΔΔCt^ method [48]. The first three stages of the cowpea weevil are too small. Here, we used the 4th instar larval stage to extract and analyze the HIF-1 gene expression.

### 4.7. Monitoring Eclosion of Cowpea Weevils from Beans Treated with HIF-1 Inhibitors

Five different small-molecule compounds classified as HIF-1 inhibitors were ordered from Solarbio Life Sciences. They are 2-methoxyoestradiol (2ME2), vincristine (VCR), camptothecin (CPT), topotecan (TPT), and paclitaxel (PTX). For each compound, 800 mung beans were mixed thoroughly with 100 mg of the compound in powder form for surface coating. In total, 10 female and 10 male young adult weevils within 24 h of their hatching were then introduced to inoculate these compound-treated beans. 288 beans with a single egg laid on each bean were selected and placed in 96-well plates to be incubated at constant 30 °C for consecutive 45 days with daily recording on the number of adults with successful hatching. The observation duration of 45 days was chosen to accommodate the possibility of a severely delayed development cycle. Same procedure was repeated to study the effect induced by hypothermia where the incubation temperature was lowered from 30 °C to 4 °C only for 24 h between the 15^th^ and 16^th^ day and back to 30 °C afterwards. Control experiments, i.e., without compound treatment for beans, were also performed in parallel with the same protocol outlined above.

## Data Availability

Not applicable.

## Supplementary Materials

The following are available online at https://www.mdpi.com/article/10.3390/pathogens10060704/s1, Table S1. Survival rate of cowpea weevils under different inhibitor treatments; Table S2. Developmental duration of cowpea weevil under low-temperature treatment; Figure S1. Morphological characteristics of three different subphases of egg development of cowpea weevil.
