# Supplementary material for: Insights into Temperature and Hypoxia Tolerance in Cowpea Weevil via HIF-1
Source: Pathogens. 2021 Jun 5;10(6):704. doi: 10.3390/pathogens10060704 (PMC8228136; doi:10.3390/pathogens10060704)
Supplement: Supplementary file 1 [file pathogens-10-00704-s001.zip › pathogens-1216136-SI.pdf]

Supporting information

Insights into temperature and hypoxia tolerance in cowpea weevil via HIF-1

Qin Liu[1], Zhichao Liu[3], Zhipeng Gao[1], Guanjun Chen[1], Changyan Liu[4], Zhenghuang Wan[4], Chanyou Chen[1], Chen Zeng[5], Yunjie Zhao[2]*, Lei Pan[1]*

[1]School of Life Sciences, Jianghan University, Wuhan, 430056, China.

[2]Institute of Biophysics and Department of Physics, Central China Normal University, Wuhan, 430079, China.

[3]School of Biological Information, Chongqing University of Posts and Telecommunications, Chongqing, 400065, China.

[4]Institute of Food Crop, Hubei Academy of Agricultural Sciences, Wuhan, 430064, China.

[5]Department of Physics, The George Washington University, Washington, DC, 20052, USA.

*Correspondence:
Yunjie Zhao Email: yjzhaowh@mail.ccnu.edu.cn
Lei Pan Email: leipan@jhun.edu.cn

Table S1. Survival rate of cowpea weevils, i.e., the fraction of eggs that developed into adults, under different inhibitor treatments. Treatment (4 °C) denotes the experimental setting where the incubation temperature of 30 °C was temporarily dropped to 4 °C for 24 h in the 16th day of the development cycle.

**Treatment (30 °C)**	**Number of initial eggs**	**Survival rate (%)**	**Developmental duration (average±sd)**
Control	288	84.0	20 – 34 (23.4±2.0)
2ME2	288	73.3	23 – 36 (25.6±2.6)
TPT	288	67.7	21 – 32 (23.4±2.0)
PTX	288	20.8	22 – 39 (30.2±5.3)
VCR	288	17.7	23 – 39 (28.8±4.7)
CPT	288	3.5	22 – 36 (28.3±4.9)
**Treatment (4 °C)**	**Number of initial eggs**	**Survival rate (%)**	**Developmental duration (average±sd)**
Control	288	70.8	21 – 35 (24.4±2.5)
2ME2	288	71.9	23 – 32 (26.1±1.9)
TPT	288	63.9	21 – 37 (25.1±3.0)
PTX	288	26.7	22 – 39 (30.9±4.6)
VCR	288	15.6	24 – 39 (30.9±4.8)
CPT	288	2.7	27 – 37 (32.6±3.7)

Note: 2-methoxyoestradiol (2ME2), vincristine (VCR), camptothecin (CPT), topotecan (TPT), and paclitaxel (PTX).

Table S2. Developmental duration of cowpea weevil under low-temperature treatment

Temperature（℃）	Time（h）	Egg duration（d）	Larval duration（d）	Pupae duration（d）
-20	0.5	5.51 ± 0.14	16.01 ± 0.08	22.51 ± 0.35
	1	5.63 ± 0.11	16.07 ± 0.19	nt
	2	nt	16.59 ± 0.16	nt
	4	nt	16.31 ± 0.12	nt
4	4	5.33 ± 0.11	16.08 ± 0.07	22.44 ± 0.12
	8	5.68 ± 0.06	16.19 ± 0.34	22.72 ± 0.41
	16	5.87 ± 0.07	16.18 ± 0.42	22.63 ± 0.23
	32	5.93 ± 0.12	16.34 ± 0.29	22.86 ± 0.21
	64	6.64 ± 0.24	16.69 ± 0.20	22.43 ± 0.36
	128	7.22 ± 0.15	17.16 ± 0.28	23.01 ± 0.11
15	4	5.68 ± 0.17	16.13 ± 0.13	21.13 ± 0.32
	8	5.92 ± 0.05	16.14 ± 0.80	22.41 ± 0.79
	16	5.97 ± 0.21	16.55 ± 0.13	22.45 ± 0.23
	32	6.72 ± 0.32	16.71 ± 0.28	22.85 ± 0.45
	64	7.82 ± 0.05	17.06 ± 0.35	22.37 ± 0.14
30	b	5.05 ± 0.02	14.42 ± 0.90	21.62 ± 0.72

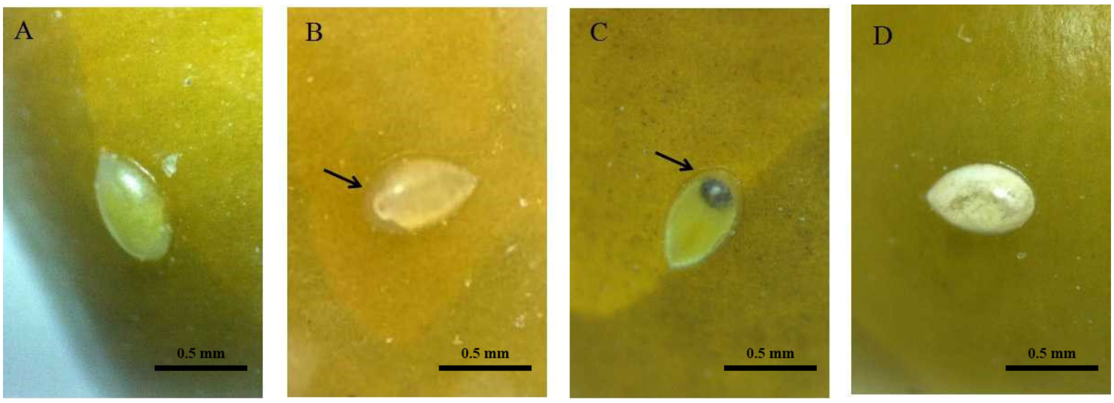

Figure S1. Morphological characteristics of three different subphases of egg development of cowpea weevil. (A) Early subphase; (B) Middle subphase; (C) Late subphase (start), also called black-headed phase; (D) Late subphase (end) when the larva has burrowed into the bean with a white and opaque eggshell from the frass deposits.
